# mRNA vaccine in cancer therapy: Current advance and future outlook

**DOI:** 10.1002/ctm2.1384

**Published:** 2023-08-23

**Authors:** Youhuai Li, Mina Wang, Xueqiang Peng, Yingying Yang, Qishuang Chen, Jiaxing Liu, Qing She, Jichao Tan, Chuyuan Lou, Zehuan Liao, Xuexin Li

**Affiliations:** ^1^ Department of Breast Surgery Baoji Municipal Central Hospital Weibin District Baoji Shaanxi China; ^2^ Graduate School Beijing University of Chinese Medicine Beijing China; ^3^ Department of Acupuncture and Moxibustion Beijing Hospital of Traditional Chinese Medicine Capital Medical University Beijing Key Laboratory of Acupuncture Neuromodulation Beijing China; ^4^ Department of General Surgery The Fourth Affiliated Hospital China Medical University Shenyang China; ^5^ Clinical Research Center Shanghai Key Laboratory of Maternal Fetal Medicine Shanghai Institute of Maternal‐Fetal Medicine and Gynecologic Oncology Shanghai First Maternity and Infant Hospital School of Medicine Tongji University Shanghai China; ^6^ Department of Ophthalmology Xi'an People's Hospital (Xi'an Fourth Hospital) Xi'an Shaanxi China; ^7^ School of Biological Sciences Nanyang Technological University Singapore Singapore; ^8^ Department of Microbiology, Tumor and Cell Biology (MTC) Karolinska Institutet Sweden; ^9^ Department of Medical Biochemistry and Biophysics (MBB) Karolinska Institutet Biomedicum Stockholm Sweden

**Keywords:** cancer, cancer vaccine, immunology, immunotherapy, mRNA vaccine

## Abstract

Messenger ribonucleic acid (mRNA) vaccines are a relatively new class of vaccines that have shown great promise in the immunotherapy of a wide variety of infectious diseases and cancer. In the past 2 years, SARS‐CoV‐2 mRNA vaccines have contributed tremendously against SARS‐CoV2, which has prompted the arrival of the mRNA vaccine research boom, especially in the research of cancer vaccines. Compared with conventional cancer vaccines, mRNA vaccines have significant advantages, including efficient production of protective immune responses, relatively low side effects and lower cost of acquisition. In this review, we elaborated on the development of cancer vaccines and mRNA cancer vaccines, as well as the potential biological mechanisms of mRNA cancer vaccines and the latest progress in various tumour treatments, and discussed the challenges and future directions for the field.

## Graphical abstract

- Messenger ribonucleic acid vaccines deliver tumour antigens to antigen‐presenting cells (APCs) for the presentation to immune cells (CD8^+^ T, CD4^+^ T) via major histocompatibility complex classes I and II.
- Innate immune activity can also be induced through APC‐expressed pattern recognition receptors.
- Antigen receptors (chimeric antigen receptors and T cell receptors) can be introduced into lymphocytes.
- Expressions of immunomodulatory proteins in different cell types, such as other monoclonal antibody formats, chemokine receptors, costimulatory ligands and Toll‐like receptors, etc are illustrated.

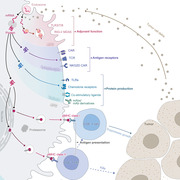

## 1 INTRODUCTION

As one of the most dreaded diseases in human beings, the emergence of immunotherapy has revolutionized the management of multiple cancer types and shed light on cancer patients. Cancer immunotherapy was determined as ‘breakthrough of the year’ in 2013 due to the successful translation from fundamental research into clinical treatments, as well as the simple yet elegant approach of it.^1^ The role of cancer immunotherapy was predominantly underrated in the 20th century on account of the lack of a known mechanism in the field, meanwhile, the routes to develop appropriate clinical schemes are full of twists and turns.

William B. Coley, an American surgeon who was universally acknowledged as the pioneer of immunotherapy, first ventured to manipulate the immune system to tackle patients with inoperable cancer in the late 19th century.^2^, ^3^ By injecting a mixture of live and inactivated bacteria (called ‘Coley's toxin’) such as *Streptococcus pyogenes* and *Serratia marcescens*, Coley found induction of intensive immune response in cancer patients,^4^ leading to remission of tumour progression. This trial was deemed the first documented anti‐cancer immunotherapy intervention in history.^5^ However, due to the insufficient knowledge of complex humoral immune system and notable advances in traditional treatments at that time, the next revolutionary wave in cancer immunotherapy came late in the 21st century until the consensus was made,^6^ when people realized that boosting innate defences to get rid of malignant cells is a monumental milestone for cancer treatment. In recent years, cancer vaccines have attracted tremendous attention due to the striking signs of progress in the field.^7^

The cancer vaccine was first developed in 1988. Mitchell et al. immunized melanoma patients with allogeneic melanoma lysate, and successfully induced anti‐melanoma immune response in hundreds of patients.^8^ Subsequently, thanks to the discovery of tumour antigens (TAs)—antigenic substances overexpressed in the tumour tissue that regulate tumour initiation, progression and metastasis, scientists are enabling more chances to target tumour cells with the potential candidates for use in cancer therapy.^9^, ^10^, ^11^, ^12^, ^13^, ^14^ In the past 20 years, several methods of antigen delivery have been presented,^15^ some of which have shown strong anti‐tumour immune responses and clinical responses in cancer patients.

Technically, the development of tumour vaccines is largely similar to vaccines for infectious diseases,^16^ including whole‐cell vaccines, DNA and messenger ribonucleic acid (mRNA) vaccines, antigen vaccines and dendritic cell (DC) vaccines.^17^ So far, hundreds of clinical trials have been conducted and demonstrated the promise and challenges posed by therapeutic vaccines (Table 1). Unlike traditional surgery, chemotherapy or radiotherapy, therapeutic cancer vaccines are considered to specifically activate the immune system and target tumour cells,^18^, ^19^, ^20^ leading to higher response rates and better quality of life. Collectively, cancer vaccines are conducive to blocking tumour growth, recurrence or metastasis and recent progress in immuno‐oncology research has exploited an unprecedented avenue for the emergence of vaccine strategies. In recent years, mRNA vaccines have progressed rapidly in the field of tumour biotherapy as an important type of tumour vaccine due to the advantages of low toxicity, fast production and a wide variety of encoded antigens. Specifically, exogenous SAMs encoding tumour‐specific antigens are introduced into somatic cells to induce immune responses by synthesizing antigens and thereby achieving specific killing of tumour cells. The promise and advances of mRNA vaccines in tumour biotherapy have been well established in recent researches. This review summarizes and discusses the advantages and limitations of mRNA vaccines in detail.

**TABLE 1:** mRNA vaccine for cancer therapy clinical trials.

Cancer type	Clinical Trials.gov identifier	Phase	Vaccine type	Sponsors	Investigators	Study start date
Non‐Small Cell Lung Cancer	NCT03908671	NA	Personalized mRNA tumour vaccine	Stemirna Therapeutics	The First Affiliated Hospital of Zhengzhou University	9 April 2019
Gastric Adenocarcinoma, Pancreatic Adenocarcinoma, Colorectal Adenocarcinoma	NCT03468244	NA	Personalized mRNA vaccine	Changhai Hospital, other institutions (OIs)	Xianbao Zhan, Dr. Chanhai Hospital	1 May2018
	NCT04163094	1	A liposome‐formulated mRNA vaccine	University Medical Center Groningen, OIs	H. W. Nijman, MD/PhD University Medical Center Groningen, UMCG More information	14 November, 2019
	NCT01334047	1, 2	DC‐006 vaccine	Steinar Aamdal	Steinar Aamdal, MD, PhD Prof. Oslo University Hospital—Norwegian Radium Hospital More information	12 April 2011
	NCT00929019	1, 2	Autologous dendritic cells electroporated with mRNA	Radboud University, OIs	Cornelis JA Punt, Prof. MD Radboud University Nijmegen Medical Centre, Department of Medical Oncology	26 June, 2009
	NCT00243529	1, 2	Autologous dendritic cell vaccine	Radboud University, OIs	Prof. C.J.A. Punt, MD, PhD Radboud University Nijmegen Medical Center, OIs	24 October, 2005
	NCT00961844	1, 2	Dendritic cells—transfected with hTERT‐, survivin‐ and tumour cell‐derived mRNA + ex vivo T cell expansion and reinfusion	Steinar Aamdal	Steinar Aamdal, MD PhD Prof. Oslo University Hospital—Norwegian Radium Hospital	19 August 2009
	NCT03897881	2	mRNA‐4157	ModernaTX, Inc., OIs	NA	1 April 2019
Breast Cancer	NCT00978913	1	DC‐vaccine	Inge Marie Svane	Inge Marie Svane, Prof. MD Department of Oncology, Herlev University Hospital, Herlev Ringvej 75,2730 Herlev	17 September 2009
	NCT01456104	1	Langerhans‐type dendritic cells (a.k.a. Langerhans cells or LCs)	Memorial Sloan Kettering Cancer Center, OIs	James Young, MD Memorial Sloan Kettering Cancer Center	20 October 2011
Colon Cancer Gastrointestinal Cancer	NCT03480152	1, 2	Messenger RNA (mRNA)‐based, personalized cancer vaccine	National Cancer Institute (NCI), OIs	Steven A Rosenberg, MD National Cancer Institute (NCI)	29 March 2018
	NCT00940004	1, 2	DC‐vaccine	Radboud University	Radboud University Nijmegen Medical Centre	15 July 2009
	NCT01066390	1	TriMix‐DC	Bart Neyns, OIs	UZ Brussel	10 February 2010
	NCT00204516	1, 2	mRNA coding for melanoma‐associated antigens	University Hospital Tuebingen, OIs	Claus Garbe, Prof. Dr. University of Tuebingen, Department of Dermatology	20 September, 2005
	NCT00087373	2	Recombinant fowlpox‐TRICOM vaccine	National Cancer Institute (NCI)	University of Chicago	12 July 2004
	NCT00204607	1, 2	NA	University Hospital Tuebingen	Claus Garbe, Prof. Dr. University of Tuebingen, Department of dermatology	20 September, 2005
	NCT01153113	Withdrawn	hTERT mRNA DC	NA	NA	29 June 2010
	NCT00514189	1	Autologous dendritic cells	National Cancer Institute (NCI), OIs	UT MD Anderson Cancer Center	9 August 2007
	NCT01278914	1, 2	Dendritic cells (DC) prostate vaccine	Oslo University Hospital	NA	19 January 2011
	NCT01446731	2	DC‐vaccine	Inge Marie Svane	Per Kongsted, MD CCIT/Department of Oncology, Herlev Hospital	5 October 2011
	NCT02140138	2	CV9104	CureVac AG	Nationales Zentrum für Tumorerkrankungen, Medizinische Onkologie	16 May 2014
Neoplasm Metastases	NCT02808416	NA	Personalized cellular vaccine	Guangdong 999 Brain Hospital, OIs	Jian Zhang, MD Guangdong 999 Brain Hospital	21 June 2016
Glioblastoma	NCT00846456	1, 2	Dendritic cell vaccine with mRNA from tumour stem cells	Oslo University Hospital	Steinar Aamdal, MD, PhD Department of Clinical Cancer Research, Rikshospitalet, OIs	18 February 2009
	NCT01885702	1, 2	DC‐vaccine	Radboud University	Radboud University Nijmegen Medical Centre	25 June 2013
	NCT00228189	1, 2	CEA‐loaded dendritic cell vaccine	Radboud University	Prof. Dr. C.J.A. Punt, MD,PhD Radboud University Nijmegen Medical Center, dept. of Medical Oncology	28 September 2005
Renal Cell CarcinomaSarcomas	NCT01291420	1, 2	DC‐vaccine	University Hospital, Antwerp	NA	8 February 2011
	NCT03548571	2, 3	Dendritic cell immunization	Oslo University Hospital	Oslo University Hospital	7 June 2018
	NCT02808364	1	Personalized cellular vaccine	Beijing Tricision Biotherapeutics Inc, OIs	Guangdong 999 Brain Hospital	31 October 2017
Malignant Glioma Astrocytoma	NCT02529072	1	DC‐vaccine	Gary Archer Ph.D., OIs	Duke University Medical Center	12 August 2015
Glioblastoma Multiforme Malignant Glioma	NCT02465268	2	pp65‐shLAMP DC with GM‐CSF, unpulsed PBMC and saline	Immunomic Therapeutics, Inc., OIs	University of Florida	8 June 2015
	NCT02649582	1, 2	DC‐vaccine	University Hospital, Antwerp	University Hospital, Antwerp	7 January 2016
	NCT04911621	1, 2	DC‐vaccine	University Hospital, Antwerp, OIs	University Hospital, Antwerp	3 June 2021
	NCT04573140	1	Autologous total tumour mRNA and pp65 full length (fl) lysosomal‐associated membrane protein (LAMP)	University of Florida, OIs	UF Health	5 October 2020
	NCT01686334	2	DC‐vaccine	Zwi Berneman, OIs	Zwi Berneman, MD, PhD University Hospital, Antwerp, OIs	18 September 2012
Chronic Myeloid Leukemia Multiple Myeloma	NCT00965224	2	DC‐vaccine	University Hospital Antwerp, OIs	University Hospital, Antwerp	25 August 2009
	NCT01734304	1, 2	DC‐vaccine	Ludwig‐Maximilians—University of Munich	Hospital of the University of Munich	27 November 2012
	NCT00834002	1	DC‐vaccine	University Hospital, Antwerp	Zwi Berneman, MD, PHD University Hospital, Antwerp, OIs	2 February 2009
	NCT00923312	1, 2	CV9201	CureVac AG	RWTH Aachen, OIs	18 June 2009
Ewings SarcomaLiver Cancer	NCT01061840	1	Vigil^™^	Gradalis, Inc.	Minal Barve, MD Mary Crowley Cancer Research Centers	6 December 2018
	NCT00626483	1	RNA‐loaded dendritic cell vaccine	Gary Archer Ph.D., OIs	Mustafa Khasraw, MD Duke University	29 February 2008
	NCT00639639	1	NA	Gary Archer Ph.D.,OIs	Katherine Peters, MD, PhD Duke University Medical Center	20 March 2008
	NCT01995708	1	CT7, MAGE‐A3 and WT1 mRNA‐electroporated Langerhans cells ( LCs)	Memorial Sloan Kettering Cancer Center	David Chung, MD, PhD Memorial Sloan Kettering Cancer Center	26 November 2013
	NCT02649829	1, 2	DC‐vaccine	Kom Op Tegen Kanker, OIs	Antwerp University Hospital, OIs	8 January 2016
	NCT02693236	1, 2	Adenovirus‐transfected autologous DC vaccine plus CIK cells	Affiliated Hospital to Academy of Military Medical Sciences	NA	26 February 2016
	NCT00890032	1	BTSC mRNA‐loaded DCs	National Cancer Institute (NCI),OIs	Gordana Vlahovic, MD Duke University	29 April 2009

## 2 CANCER VACCINE: STARTING FROM THE BASICS

### 2.1 Fundamentals of the cancer vaccine

The immune system is a sophisticated network of cells and proteins. Technically, it offers the body protection, so‐called immunity, which is achieved through the presence of antibodies or cell‐mediated immunity to diseases in an individual's immune system, against disease.^21^ Among multiple mechanisms of immune response, vaccines are vital to boost the body's immune system, such as by stimulating the production of antibodies against foreign pathogens. Over the past decades, tumour antigen has been extensively exploited and proved to be of great significance in cancer immunotherapy, largely promoting the discovery of cancer vaccines, and eliciting long‐lasting and effective immune responses.^22^ In general, cancer vaccines can be classified based on their applications and functions. Classical cancer preventive vaccines target the viruses that can cause certain cancers,^23^ whereas therapeutic vaccines can stimulate immune responses against existing tumours. Optimally speaking, 15% of cancer cases could be blocked by fighting infections and it turned out that infection is considered a causative factor in an estimated one in four cancers.^24^ Theoretically, it is easier, cheaper and more effective to vaccinate against viruses that lead to cancer than it is to treat cancer with a cancer therapeutic vaccine. However, due to poor immunogenicity and limited safety, only two preventive cancer vaccines have successfully passed clinical trials and have been approved by the U.S. food and drug administration (FDA).^25^, ^26^

### 2.2 Vaccines for cancer prevention‐targeting oncogenic virus

Oncoviruses are implicated in approximately 12% of all human cancers.^27^ The evidence that chronic infections with hepatitis B virus (HBV) and high‐risk human papillomavirus (HPVs) are important caustic factors for hepatocellular carcinoma and cervical cancer, respectively,^28^, ^29^ has been underpinned solidly over the past three to four decades. The HBV vaccine is known as the first ‘anti‐cancer’ vaccine, which was first implemented for HBV in 1982, with the development of a highly innovative, novel prophylactic vaccination approach, widespread HBV immunization of neonates has exerted a great impact on the incidence of chronic HBV infection in children 5 years of age.^30^

Additionally, HPV is a universal public health problem with high rates of cervical cancer, it was confirmed as a causal agent of cervical cancer by Harald Zur Hausen,^31^ a German virologist, who first isolated HPV strains in cervical cancer tumours in the 1980s, this theory led to the completion of the first human trial for the HPV vaccine, named Gardasil in 2006.^32^ Since then, two further vaccines have been approved: Cervarixin 2007 and Gardasil 9 in 2014.^33^, ^34^ As of June 2020, more than 100 countries worldwide have included HPV vaccine in their national immunization programs as part of their regular vaccine schedule. Nonetheless, many oncogenic DNA viruses have been under investigation.^35^

### 2.3 Vaccines for cancer treatment: New hope in the cancer immunotherapy

Unlike prophylactic vaccines which are exploited against viruses, the therapeutic cancer vaccine aims to operate the immune system to mount an attack against cancer cells or tissues in the body.^36^ Technically, the precondition for the development of a therapeutic cancer vaccine depends on the presence of the tumor‐associated antigen (TAA) or tumour‐specific antigens (TSAs).^37^ To date, most cancer vaccines have targeted TAAs, which are self‐proteins that are abnormally expressed by cancer cells, nevertheless, obstacles have to be overcome before developing vaccines against TAAs. For example, immune cells may recognize TAAs as self‐antigen, remove them from the immune repertoire, leading to failure in immune responses.^38^, ^39^ TSAs represent antigens that are specific to tumour cells, and have aroused extensive attention due to their specific reaction with immune cells. However, due to uniqueness of neoantigens to each patient and tumour type, further optimisation needs to be conducted for the sake of reducing the cost and complexity of TSA vaccine.^40^

Although major challenges in terms of efficacy and safety are still existing, diverse therapeutic vaccination strategies have been under development in the pre‐clinical stage or evaluated in clinical trials. Based on the platforms used in vaccine development, therapeutic vaccines are classified into various major categories.^41^ Among those, mRNA vaccines are a promising alternative to conventional vaccine approaches.^17^ Up to now, multiple mRNA cancer vaccines have been employed in various clinical trials, leading to the notion that this strategy can be broadly applicable to cancer vaccines.^42^

## 3 mRNA CANCER VACCINES: HISTORY AND RECENT ADVANCES

As aforementioned, cancer and infectious diseases are the two most common challenges humankind is faced with currently, despite multiple in‐progress research and treatment measures, satisfactory results are yet to be achieved. The emergence of new pandemics like COVID‐19, Ebola, Zika, HIV and measles has necessitated the need for finding a new approach or new technique unlike before to overcome the crisis in healthcare and for the betterment of human life.^43^, ^44^, ^45^, ^46^ A dormant technique that was considered almost impossible to be implemented in the 19th century was ventured again in the last decade and reinstalled confidence in the form of nucleic acid encoded therapeutics. Recent advances in research have given us deeper knowledge of human genetics and have created an avenue for mRNA as a promising treatment protocol for infectious diseases and cancer.^17^, ^47^ The human genome contains nearly 21306 genes that code for proteins.^48^ The genes in the DNA are transcribed into mRNA inside the nucleus of a cell. The transcribed mRNA is a highly unstable molecule that carries information from genes in the nucleus to ribosomes in the cytoplasm for protein synthesis.^17^ So, mRNA is the intermediate of the protein synthesis process in our body. Given that mRNA can be translated into proteins, this principle can be utilized to produce any protein like defective enzymes, hormones, antibodies, cell structure proteins and foreign antigens.^49^ As a result, mRNA vaccines are eye‐catching as they are easy to produce, manipulate and deliver into cells.^50^, ^51^ In comparison, other techniques of genomic/genetic engineering need manipulation of the genome that might lead to undesirable effects such as off targeting. These effects can be minimized by using mRNA vaccines. Of note, in cancer, mRNA is used to induce the immune system to destroy tumour‐associated antigens and growth factors.^52^, ^53^, ^54^ The current COVID‐19 pandemic has necessitated the need for the development of a new vaccine and has accelerated research developments in mRNA therapeutics at a tremendous pace.^55^, ^56^, ^57^, ^58^, ^59^

Although vaccinology has made outstanding achievements in preventing various diseases, there are still major hurdles to the development of vaccines against cancer, emphasizing the necessity to develop a more effective and functional vaccine platform.^60^ However, due to mRNA instability, high innate immunogenicity and in vivo delivery efficiency, the study of mRNA vaccines once lagged behind the research of DNA and protein‐based vaccine development until technological innovations and research investments recognized mRNA as a versatile tool for the development of new innovative therapeutics, especially in the field of vaccinology in the past decades.^42^

### 3.1 History

**FIGURE 1:**
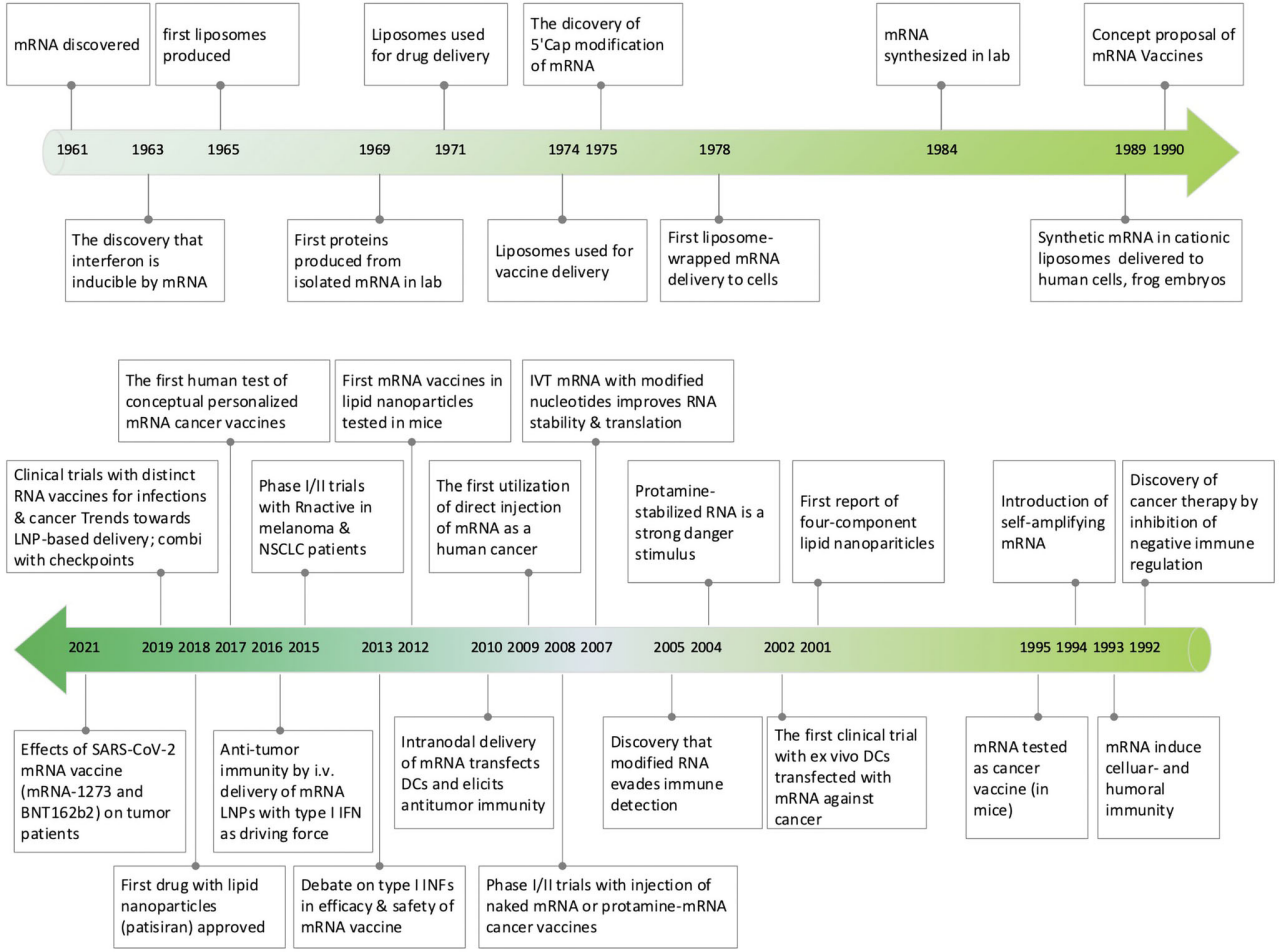
Timeline: Breakthroughs in mRNA vaccine research and its progress in cancer therapy.

The study of mRNA structure and function has been fascinating since the first human discovery of mRNA in 1961.^61^ Notably, the first in vitro translation of isolated mRNA was achieved by humans in 1969, which supported researchers in synthesizing specific proteins in vitro.^62^ Also, with the rapid advancement of vaccine delivery systems, researchers developed liposome‐encapsulated mRNA delivery systems in 1978, which made it possible to regulate proteins within target cells.^63^, ^64^ This was followed by Jirowski et al. (1992) who successfully used vasopressin mRNA to treat diabetes in mice insidiously. Most importantly, in 1990 Wolff et al. used mRNA‐encoded proteins for vaccination in mice for the first time.^65^ Jirowski et al. followed Wolff with the successful use of pressin mRNA for the treatment of diabetes in mice.^66^ These results widened the scope of mRNA‐based therapeutics. In 1995, in tumour immunotherapy, researchers confirmed the potential of mRNA applications with the first introduction of mRNA‐encoded luciferase and carcinoembryonic antigen vaccines with good results.^67^ However, this technology has gradually been clinically validated in recent years with breakthroughs in technical difficulties related to mRNA structural stability and delivery methods. Into the 2000s, mRNA vaccines entered a period of rapid development because of their unique advantages (ease and rapidity of design and detection, inherent immunogenicity, quick preparation and negligible risk of insertional mutagenesis). This was followed by a remarkable achievement in 2005 when nucleoside‐modified RNA was found to be non‐immunogenic, which brought new light to further mRNA vaccine research.^68^ Prostate cancer vaccines entered clinical trials in 2015.^69^ In 2019, the variable splicing of SAMs for cancer immunotherapy was found by researcheres.^70^ Importantly, in 2020 and 2021, the medical value of mRNA vaccines was realized with the approval of two mRNA vaccines against COVID‐19, Comirnaty (BNT162b2) and Spikevax (mRNA‐1273)^71^, ^72^ (Figure 1). Nowadays, mRNA vaccines are often reported, and in the future, they may become one of the essential technologies for disease prevention and treatment.^19^

### 3.2 Advantages of mRNA cancer vaccines

Although the application of mRNA vaccines in cancer treatment is still nascent, numerous features of in vitro transcribed mRNA have indicated its vaccine potential, additionally, mRNA vaccines have manifested several striking advantages over peptide or DNA vaccines, which could be more effective against a wide range of cancers.

First, the development of RNA‐based vaccines is relatively faster and cheaper than conventional vaccines owing to the high yields of in vitro transcription (IVT) reactions^73^ and the advanced industrial setup which revolutionized the manufacture of mRNA and significantly reduces the cost of production to an extent.^74^ For example, in a phase 1 clinical trial in 2020, the first volunteer was administrated the COVID‐19 mRNA vaccine 10 weeks after the sequence of the viral genome revealed.^75^ As of November 2020, there were already two novel mRNA vaccines awaiting authorization as potential COVID‐19 vaccines, mRNA‐1273 from Moderna and BNT162b2 from a BioNTech/Pfizer partnership.^76^, ^77^ The faster production capability of mRNA vaccines is of great value to herald rapid control over the spread of various infectious diseases as well as multiple types of cancer.

Second, mRNA vaccines are not manufactured with pathogen particles or inactivated pathogens, the non‐infectious attribute largely decreases the risk of undesired immune responses.^42^ Furthermore, compared to DNA cancer vaccines, the administration of mRNA is through a non‐integrating platform which is only required to be present in the cytoplasm other than entering the nucleus of a cell as a DNA vaccine. This feature bypasses the risk of integrating a foreign gene into the host genome and may eliminate the additional cellular (i.e., nuclear) membrane that plasmid DNA needs to cross, consequently, there is no potential risk of infection or insertional mutagenesis. Additionally, mRNA degradation is controlled by physiological cellular processes, thanks to the progress of various modifications and delivery methods, the in vivo mRNA half‐life can be therefore designed to be under regulation, which again largely guaranteed the safety of mRNA vaccine.

**FIGURE 2:**
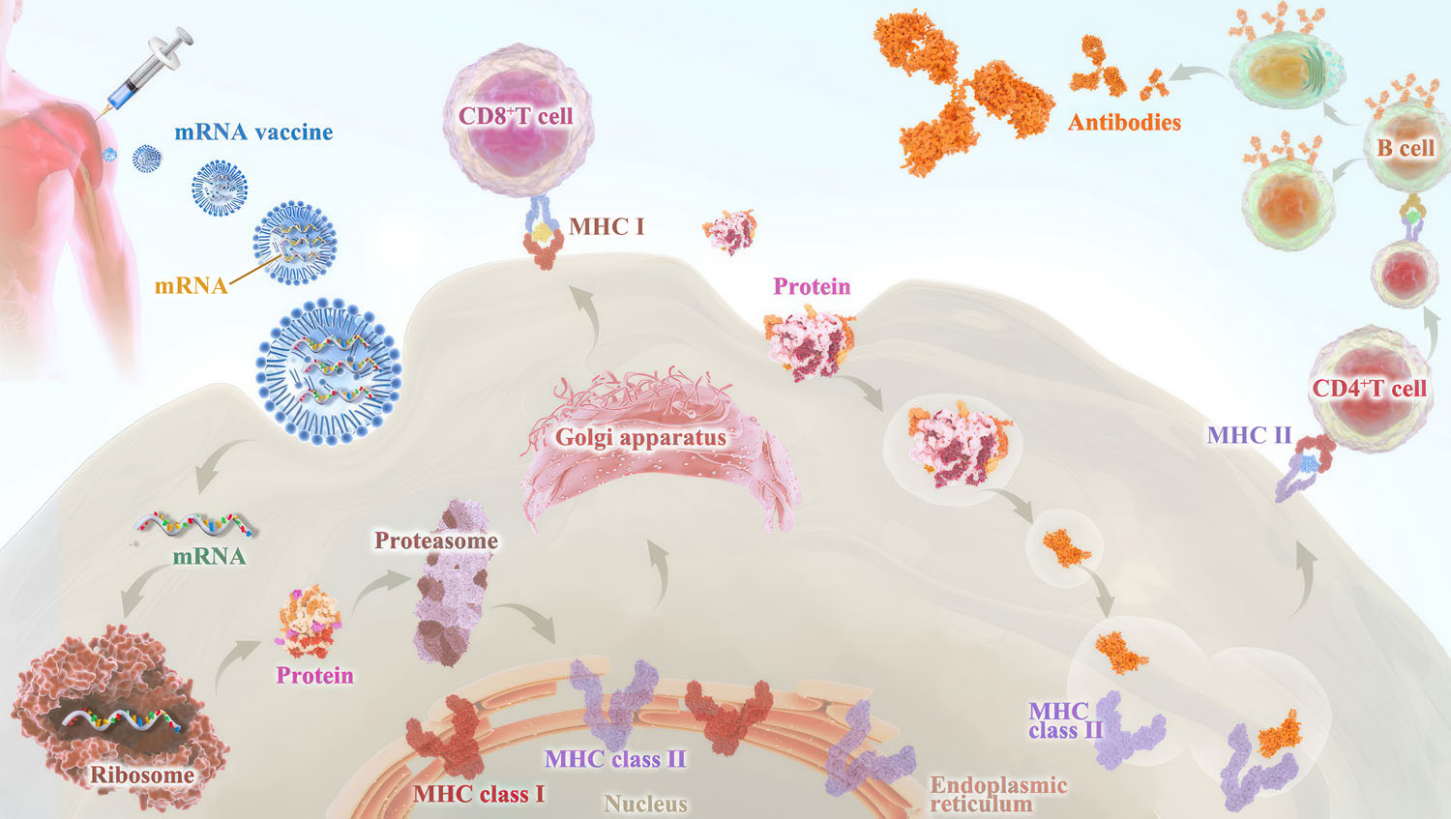
mRNA vaccines‐based immune response. Similar to virus infection, after the mRNA vaccine enters the cell through endocytosis, it transiently expresses and accumulates antigens in the cytoplasm, and then these antigens are efficiently processed into peptides and loaded on MHC‐I molecules. On the other hand, the protein expressed by mRNA can also activate the MHC‐II pathway after secretion and circulation or directly deliver the antigen from the cytoplasm to the lysosome. Therefore, it can stimulate cellular and humoral immune responses at the same time and play a better protective role.

Third, early clinical trial results have indicated that mRNA vaccines have generated a reliable immune response and are well‐tolerated by healthy individuals with relatively high efficiency (Figure 2). On the one hand, with the help of IVT, the simple procedure that allows for the template‐directed synthesis of RNA molecules of any sequence from short oligonucleotides to those of several kilobases,^78^ mRNA can be produced in a cell‐free environment by not only avoiding the contamination of microbes or the quality and safety issues in the cultured cells production^79^ but also largely improve the manufacturing efficacy, accelerating downstream purification and leading to rapid and cost‐effective manufacturing. On the other hand, current achievements in the field of in vivo delivery have successfully formulated mRNA into carrier molecules, for instance, nanoparticles, allowing rapid uptake and expression in the cytoplasm.^80^, ^81^, ^82^, ^83^, ^84^ In addition to complexing conventional mRNA into stable nanoparticles, a further improvement in RNA vaccination could be gained using self‐amplifying RNA or replicon RNA (RepRNA), which are derived from the genome backbone of an alphavirus in which the genes encoding the viral RNA replication machinery are intact, but the encoding viral structural proteins are replaced with a transgene encoding the vaccine antigen, substantially inducing strong immune responses. Precision, or personalized medicine, as a novel approach to healthcare based on each person's unique genetic makeup has provided a genomic blueprint to determine an individual's unique disease susceptibility and opened a new chapter for disease prevention and treatment.^85^, ^86^ Due to the usage of TSAs or TAA in mRNA cancer vaccine development, mRNA‐based personalized cancer vaccines have the potential to tailor therapy with the best response and highest safety margin to ensure better patient care.^87^

Technically, through next‐generation sequencing, neoepitopes could be identified on a patient's tumour cells, which guides the immune system to distinguish cancer cells from normal cells. Subsequently, the mRNA vaccine could be tailored to fit the specific antigen repertoire of each patient tumour, once injected, it has the potential to direct the patient's cells to express the selected neoepitopes and conduct clearance. In conclusion, these advantages demonstrate inherent high‐efficiency features optimal for cancer therapeutic use.^42^

### 3.3 Classification of mRNA cancer vaccines

**FIGURE 3:**
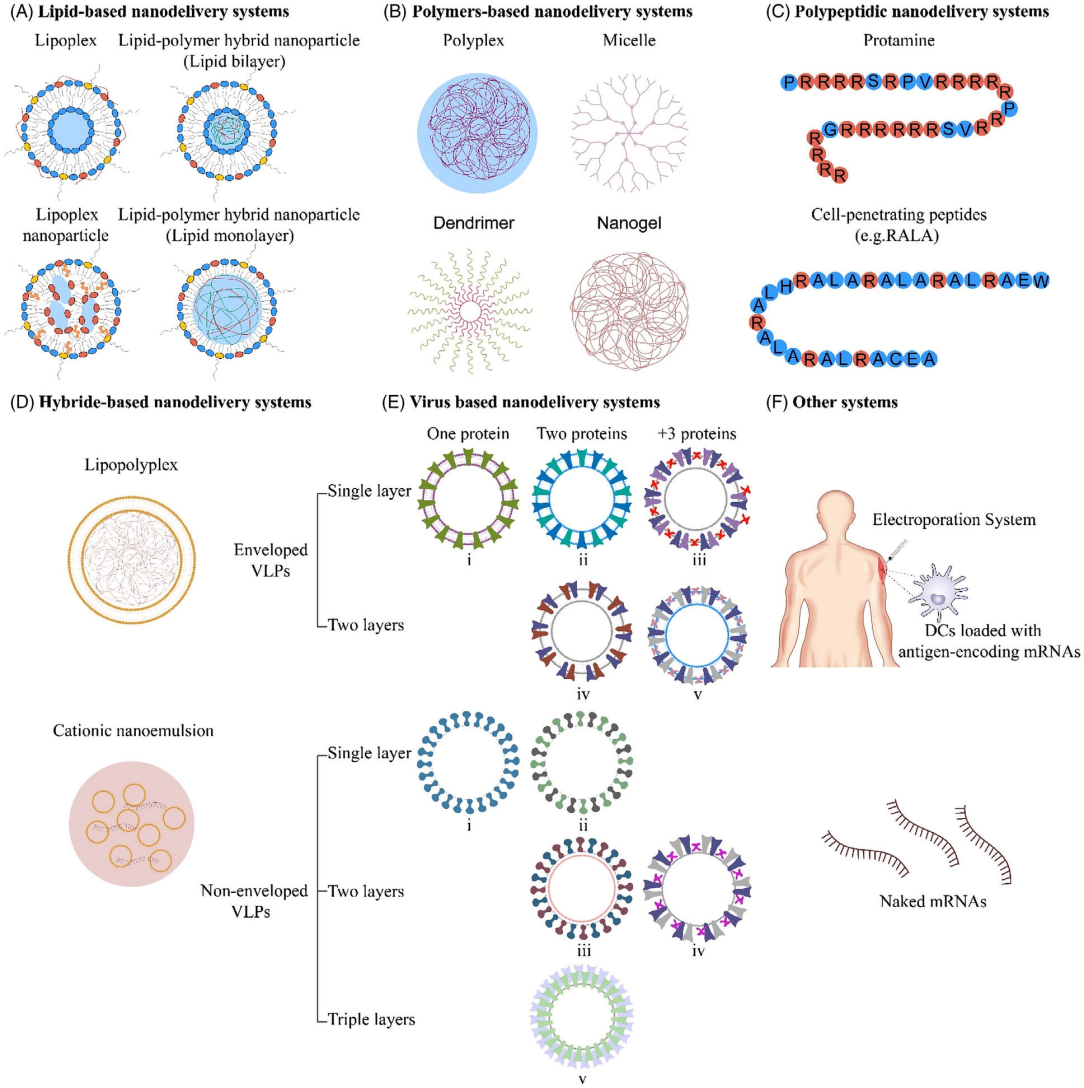
Various representative classes of mRNA‐based vaccine delivery systems. (A) Lipid‐based delivery systems. (B) Polymer‐based delivery systems. (C) Polypeptidic‐based delivery systems. (D) Hybrid‐based delivery systems. (E) Virus‐based delivery systems. (F) Other systems.

mRNA cancer vaccines are broadly classified into various categories depending on their morphology, size, physical and chemical properties. Some of them have been widely used around the world, such as lipid‐based nanodelivery systems (Figure 3A),^88^ polymer‐based nanodelivery systems (Figure 3B),^89^ polypeptidic nanodelivery systems (Figure 3C),^90^ hybrid‐based nanodelivery systems (Figure 3D),^91^ virus‐based nanodelivery systems (Figure 3E)^92^ and others (Figure 3F). Based on different vaccine characteristics, some of the well‐known classes are provided in Figure 3.

#### 3.3.1 Based on therapeutic or prophylactic property

The therapeutic vaccines are designed to induce cell‐mediated immunity to eradicate cancer cells, however, the aim of the prophylactics is to product the antibodies.^93^ Therapeutic cancer vaccines paid more attention to autoimmune complications, as the autoimmune complication is still an unsolved problem in preventive therapies. Although HPV and HBV vaccines have been approved by FDA and made a good score in cancer prevention, clinical trials of prophylactic mRNA cancer vaccine have not yet been reported. The therapeutic and prophylactic vaccines are not quite distinct from each other, sometimes prophylactic vaccines can also be used as therapeutic vaccines because they are tested effective in the reduction of the risk of clinical relapse.^94^

#### 3.3.2 Based on types of mRNA

Based on the RNA structure, there are mainly three types of mRNA cancer vaccines: non‐replicating mRNA (nrRNA) vaccine, self‐amplifying mRNA (SAM) vaccine and trans‐amplifying mRNA vaccine.

The nrRNA vaccine is a synthetic analogue of mature mRNA, whose constructs contain conventional mRNA vaccine sequences, usually including the universal 5ʹ Cap, 5ʹ untranslated regions (UTRs), an open reading frame (ORF), 3ʹUTRs and a 3ʹpoly(A) tail. The simple structure and relatively small size are the main advantages, but correspondingly, this also limits the activity and stability of nrRNA vaccine constructed in vivo, which is the major disadvantage. To enhance the durability of antigen expression, adjuvants can be added to optimise the structure of RNA molecules. TriMix is a kind of new potent adjuvant strategy with high safety, which demonstrated a superior T cell stimulatory capacity and enhancement of DCs’ maturation.^95^, ^96^ TriMix is developed by Vrije Universiteit Brussel, consisting of SAMs that encode three immune activator proteins—CD70, CD40 ligand (CD40L) and constitutively active TLR4.^97^ The application of TriMix naked mRNA in AIDS patients has achieved the desired results and aroused popular concern.^98^ For cancer therapy, De Keersmaecker B's clinical studies^99^ indicated great anti‐tumour responses in patients with terminal stage of melanoma by using TriMix electroporated together with a DC‐based mRNA vaccination.

The SAM vaccines are produced through gene engineering of positive‐stranded RNA viruses, including alphaviruses, picornaviruses, flaviviruses and so on, whose gene encoding structural proteins are replaced by antigen sequence.^100^ Compared with the nrRNA, there is an extra replication component in the construction of SAM vaccine, directing the amplification of intracellular mRNA after delivery to targeted cells.^101^ As a consequence, SAM vaccines have a higher level of antigen expression and long‐lasting efficacy with lower dosages, which is their obvious advantage.

Trans‐amplifying mRNA vaccines are a novel type which was designed by Beissert et al.^101^ It has a replicase that can amplify the RNAs ‘in trans’, which means two genes act simultaneously on different RNAs. This special structure permits a shorter length of RNA, which could reduce the difficulty of scaled‐up production and manufacturability, however, consequently adding the complexity of delivery and manufacture of two RNA drugs. Although trans‐amplifying mRNA vaccines have not been reported in clinical cancer therapy, this approach has a promising future since it could be further improved by implementing new strategies benefiting from its special structure.

#### 3.3.3 Based on route of administration

**FIGURE 4:**
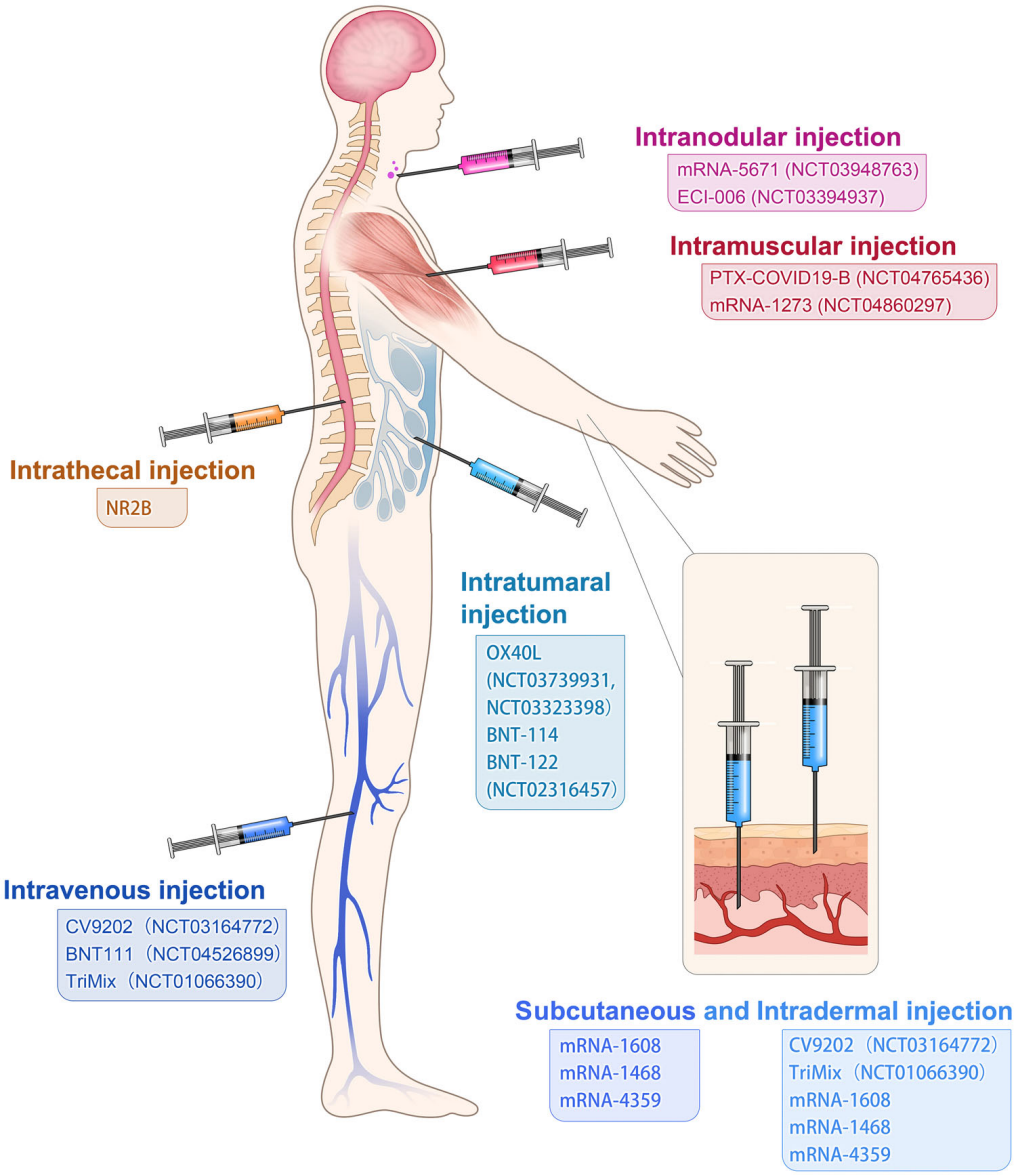
Different strategies for the administration of mRNA vaccines. 1. Intramuscular injection: LPTX‐COVID19‐B (NCT04765436 ), mRNA‐1273 (NCT04860297). 2. Intraocular injection: mRNA‐5671 (NCT03948763), ECI‐006 (NCT03394937). 3. Intravenous injection:CV9202 (NCT03164772), BNT111 (NCT04526899), TriMix (NCT01066390). 4. Intratumoural injection: OX40L (NCT03739931, NCT03323398), BNT‐114, BNT‐122 (NCT02316457). 5. Intrathecal injection: NR2B. 6. Subcutaneous and intradermal injection: Subcutaneous injection: mRNA‐1608, mRNA‐1468mRNA‐4359. Intradermal injection:CV9202 (NCT03164772), TriMix(NCT01066390), mRNA‐1608, mRNA‐1468, mRNA‐4359.

Although systematic evaluation of various administration routes in animal models or patients remains to be studied, different administration routes influence efficacy and immune‐stimulation area of mRNA cancer vaccines. At present, administration strategies of mRNA cancer vaccines include subcutaneous injection,^102^ intradermal injection,^103^ intranodular injection,^104^ intramuscular injection,^105^ intravenous injection,^106^ intratumoural injection,^107^ intrathecal injection^108^ and so on (Figure 4), which are primary but economical methods of stimulating immune responses. Local injection is considered a highly immunocompetent method, which augments local vaccine response and triggers a distal immune reaction by lymphatic transport.^103^ Intranodal or near‐nodal (into soft tissue) immunizations were usually instructed by surgical exposition of a mouse lymph node. Although intranodal injection has a complicated operation, potent T cell immunity was observed compared with other administration routes (i.d./s.c./n.n.) by Kreiter et al.^104^ Tracheal administration, targeting the lung vasculature, is usually used in pulmonary lesions, with mRNA administered as aerosol. The intrathecal injection is applied in brain lesions to help antigen presentation in cells of the central nervous system which are hard to reach. For systemic delivery, mRNA cancer vaccines are commonly administered as nanosized drug formulations and delivered intravenously, mainly targeting liver due to its abundant fenestrated capillaries.

### 3.4 Recent innovations in mRNA vaccine technologies

**FIGURE 5:**
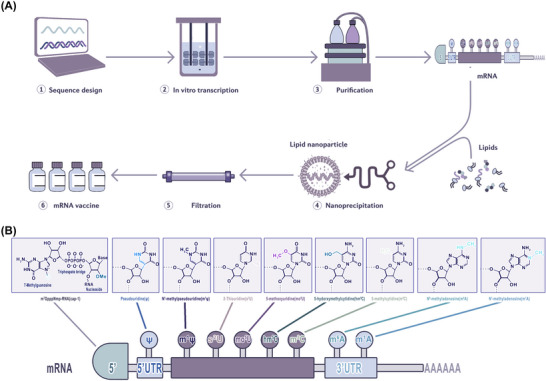
Production process of mRNA vaccines and structure of mRNA and nucleotide modifications. (A) Identification and design of a target antigen. (2) Digital sequence design. (3) Manufacturing of plasmid, mRNA and LNP. (4) Quality control (QC). (B) mRNA molecules are synthesized with a 5′‐cap one structure and chemically modified nucleotides as substitutes for natural nucleotides.

The basic structure of mRNA includes a 5′ cap, 5′ UTR, coding region, 3′UTR and a poly (A) tail.^109^ The 5′UTR or 5′ caps are crucial for producing protein efficiently, these structures may regulate cap‐dependent translation initiation.^110^, ^111^ The 3′UTR consisting of optimal poly (A) signal is required for the stability of mRNA and augmentation of protein translation.^112^, ^113^ Besides, codon optimisation is helpful to promote protein production, the abundance and stability of mRNA.^114^, ^115^ In a word, the stability and translation of mRNA determine the success of RNA vaccine production (Figure 5A). The conventional technologies, such as incorporation of modified nucleosides, optimisation of coding sequences, intranodal delivery of mRNA, ex vivo‐loaded DCs, gene gun and electroporation were a huge breakthrough for mRNA vaccine production,^116^, ^117^, ^118^ whereas these methods are complicated or costly or too hard to be used in human. Therefore, innovative technologies are needed to efficiently produce the mRNA vaccine. Recently, there are three most significant innovations in mRNA vaccine technology^119^: (1) modification of mRNA structural elements, (2) optimisation of mRNA manufacturing platform and (3) development of mRNA delivery system.

#### 3.4.1 Modification of mRNA structural elements

Given that the basic structure of mRNA plays a pivotal role in the rate of translation and half‐life of the transcript, structural improvement is of interest to mRNA vaccine design^120^ (Figure 5A). One crucial step for translation is the binding of the 5′ cap to eukaryotic initiation factor 4E (eif4e) which is the rate‐limiting step of mRNA translation. mRNA is capped enzymatically by recombinant vaccinia virus.^121^ Decapping enzymes render the mRNA molecule inactive. Synthetic cap analogues are also available, but these are not very effective. Reverse capping is a technique to increase the translational efficacy of mRNA and half‐life.^122^, ^123^ A recent study used the integration of endogenous UTRs with further de novo design to rationally engineer the UTRs of mRNA to increase protein production. Through bioinformatics analysis of endogenous gene expression and de novo design of UTRs, the most effective combination of 5′ and 3′ UTR are detected as NCA‐7d as the 5′ UTR and S27a plus a functional motif R3U as the 3′ UTR (termed NASAR UTR). The injection of TT3‐formulated receptor‐binding domain (RBD)‐encoding NASAR mRNA presented effective vaccination in mice, and intramuscular injection was five‐fold more potent than subcutaneous injection in inducing antigen‐specific antibodies.

In addition, strong vaccine antigen expression and immune response can be witnessed when using a novel RNA vaccine approach which is based on a trans‐amplifying RNA split‐vector system derived from an alphaviral self‐amplifying RNA. Then the replicase from alphaviral self‐amplifying RNA can be deleted to form a transreplicon, and transreplicon has the dose‐free properties of SAM, as a very low dose (50 ng) can effectively induce protective immune responses in mice, even when delivered as unformulated mRNA.^118^, ^124^ Since the poly (A) tail is an essential determinant for efficient translation and the lifespan of mRNA molecules, it is necessary to ensure the optimal length of the poly (A) tail,^125^, ^126^ and the incorporation of poly (A) tails at about 100 nt is ideal for mRNA therapeutics production.^127^ Convenient and stable methods of polyadenylation tend to be critical for mRNA therapeutic application. A study described a simple approach that applies type IIS restriction enzymes to generate and maintain poly (A)‐encoding DNA sequences required for IVT of mRNA. The simple approach entails repeated asymmetric cleavage with type IIS restriction enzymes, then ligation and propagation to extend the homopolymeric sequence up to approximately 100 bp in circular plasmids, which can serve as a template for mRNA transcription. Moreover, the poly (A) tail of the in vitro transcribed transcript functions in vivo as well^128^ (Figure 5B).

#### 3.4.2 Optimisation of mRNA manufacturing platform

A series of manufacturing processes should be implemented to produce therapeutic quality mRNA. Although production of the mRNA with specific quality attributes is not particularly challenging at present, a well‐established manufacturing platform is still lacking. The steps of mRNA production can be classified into upstream processing, including the enzymatic generation of mRNA, and downstream processing, involving mRNA product purification.^129^ In upstream processing, IVT enzymatic reaction used to generate mRNA is less time‐consuming than the conventional processes,^130^, ^131^ and the capping method performed during the IVT reaction shows high efficiency^132^ (Figure 5A). However, cap analogues affect the cost of production, the high price has hindered this method from being popularized, particularly during large‐scale manufacturing.^133^ Alternatively, a co‐transcriptional copping strategy termed CleanCap^®^, which does not compete with guanosine triphosphate and can add a natural 5′cap 1 construct to a specific transcription start sequence during IVT reaction, thus simplifying and reducing the cost of mRNA production.^134^ In downstream processing, removing the impurities plays a crucial role in mRNA performance. The conventional lab‐scale purification approaches comprise DNase digestion and lithium chloride (LiCl) precipitation, which cannot remove abnormal mRNA species like dsRNA and truncated RNA fragments.^118^, ^135^

The chromatography purification process is popular in pharmaceutical manufacturing. For example, size exclusion chromatography (SEC) separates molecules based on their size, but similar size impurities, such as dsRNA may be missed.^136^ The ion‐pair reverse‐phase chromatography (IPC) method can effectively remove dsRNA impurities without interfering with the process's yield. However, IPC is complicated and expensive to scale, and the use of toxic reagents prevented its generalization.^117^, ^137^, ^138^ Compared to IPC, ion‐exchange chromatography (IEC) exhibits higher binding abilities, and its scalable and cost‐effective properties make it more suitable for use in large‐scale manufacturing. Nevertheless, IEC requires a more sophisticated process.^139^, ^140^

A new cellulose‐based chromatography process, which is useful at laboratory and industrial scales, is based on the selective binding of dsRNA to cellulose in the presence of ethanol. This research claimed that more than 90% of the dsRNA contaminants can be removed from IVT mRNA samples, despite the length, coding sequence or nucleoside composition.^141^ The combination of mRNA precipitation and tangential flow filtration (TFF) technique serves as a large‐scale adaptation of general laboratory‐scale mRNA purification method. The author declared that this method can effectively remove reactants, enzymes and byproducts including prematurely aborted RNA sequences while maintaining the integrity of mRNA. Furthermore, using merely aqueous buffers as solvents rather than any caustic or flammable solvents, this method is able to be successfully carried out, indicating its effectiveness, reliability and safety in purifying mRNA^142^ (Figure 5B).

#### 3.4.3 Development of mRNA delivery systems

mRNA needs to cross the cell membrane to enter the cytosol to express specific antigens to maintain function. This is challenging due to the negative charge of both the mRNA molecules and cell membrane, relatively large size of mRNA molecules and degradability by ribonucleases existing in skin and blood.^143^ To overcome this, a number of mRNA delivery methods and mRNA delivery carriers have been explored and used currently, including naked mRNA delivery strategies and conjugation with delivery vehicles, such as lipid‐based materials, polymers or peptides.^144^, ^145^

The traditional and self‐amplifying forms of naked mRNA, injection including intramuscular/subcutaneous/intradermal/intravenous/intranasal/intramodular/intratumoural injection, can provoke the immune‐therapy response, which effectively stimulates antigen presentation and initiates immune responses^146^ (Figure 4). Moreover, a study has demonstrated that subcutaneous injection of naked mRNA in mice triggered immune responses more than mRNA nanoparticle carriers, while mRNA nanoparticle carriers perform better when administrated intranasally and intravenously.^147^ Intratumoural injection of tumour‐associated antigen mRNA is believed to be a promising vaccination method because of the induction of an appropriate immune response^148^ (Figure 4). Besides, common physical approaches like electroporation, gene gun and microneedles partially assist in improving mRNA antigen presentation.^149^ However, neither the injection of a naked mRNA delivery system nor physical ways to deliver mRNA can be applied in human patients because they are primitive and dangerous and may impact cell activities, or even cause abnormal cell death.^150^

**FIGURE 6:**
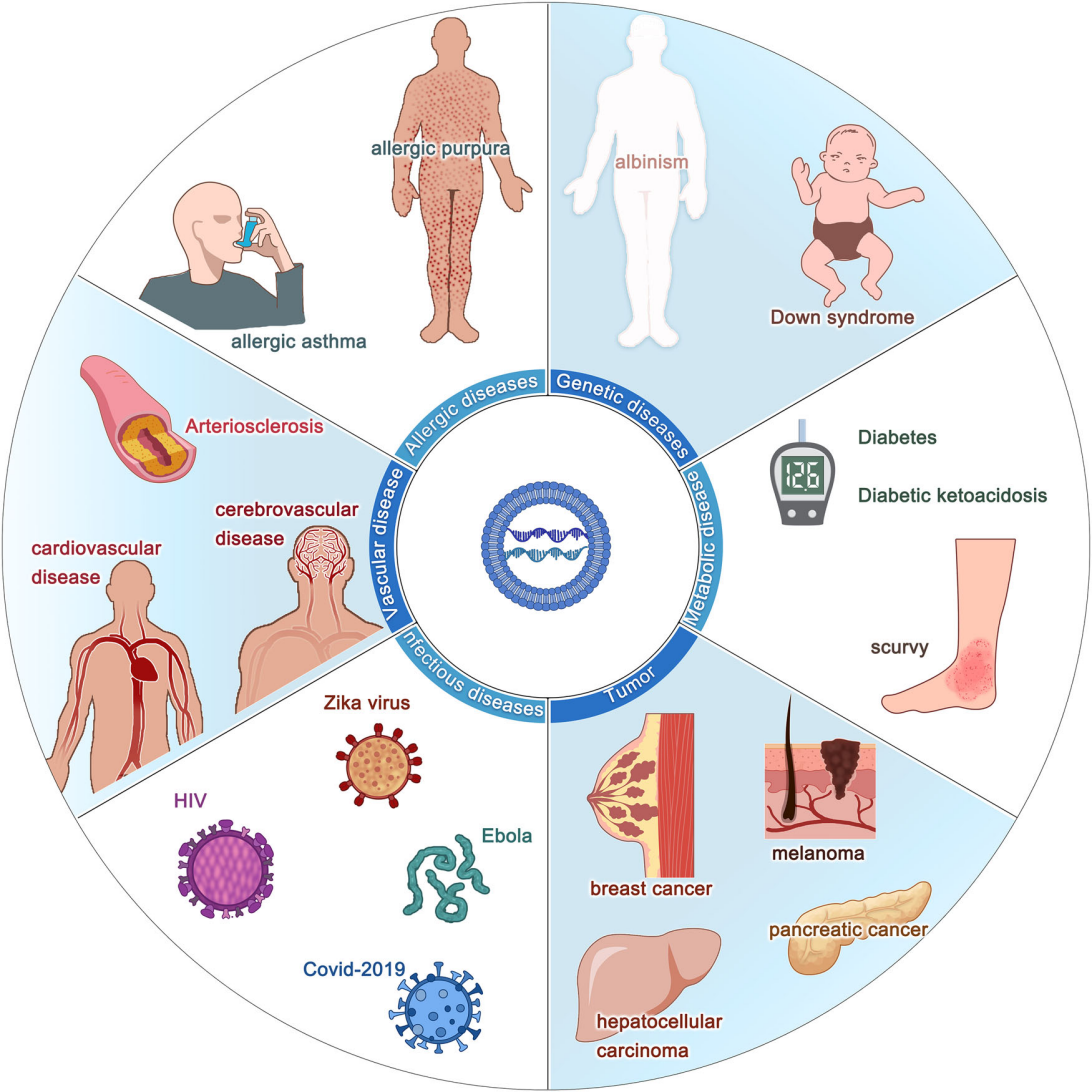
mRNA vaccines have great potential in the treatment of many diseases. 1. Metabolic disease: Diabetes, diabetic ketoacidosis, scurvy. 2. Tumour: Melanoma, breast cancer, pancreatic cancer, hepatocellular carcinoma. 3. Genetic diseases: Down syndrome, albinism. 4. Allergic diseases: allergic asthma, allergic purpura. 5. Vascular disease: arteriosclerosis, cardiovascular disease, cerebrovascular disease. 6. Infectious diseases: Zika virus, HIV, Covid‐2019, Ebola.

Liposome complexes or liposome nanoparticles (LNPs) are one of the most promising mRNA delivery tools because they can transport hydrophobic or hydrophilic molecules (e.g., small molecules, proteins and nucleic acids). Cationic liposomes which encapsulate mRNA were the first liposome delivery materials, it prevents mRNA from being degraded by RNase.^151^ pH‐responsive cationic lipids are developed as mRNA delivery vehicles to improve delivery efficacy,^152^ since other negatively charged molecules also interact with positively charged cationic lipids which can be captured by immune cells as well,^144^ LNPs, originally explored for siRNA delivery, are currently the most advanced delivery system for mRNA vaccines.^153^ The basic structure of LNPs includes an aqueous core encompassed by a lipid bilayer shell of cationic lipids, auxiliary lipids, cholesterol and polyethylene glycol, which stabilizes the particles.^154^ LNP‐mediated delivery of mRNA vaccines can induce durable, protective immune responses against multiple infectious pathogens, such as Zika^155^ and influenza,^156^ and encouraging results have been found in combatting cancer as well^157^ (Figure 6).

**FIGURE 7:**
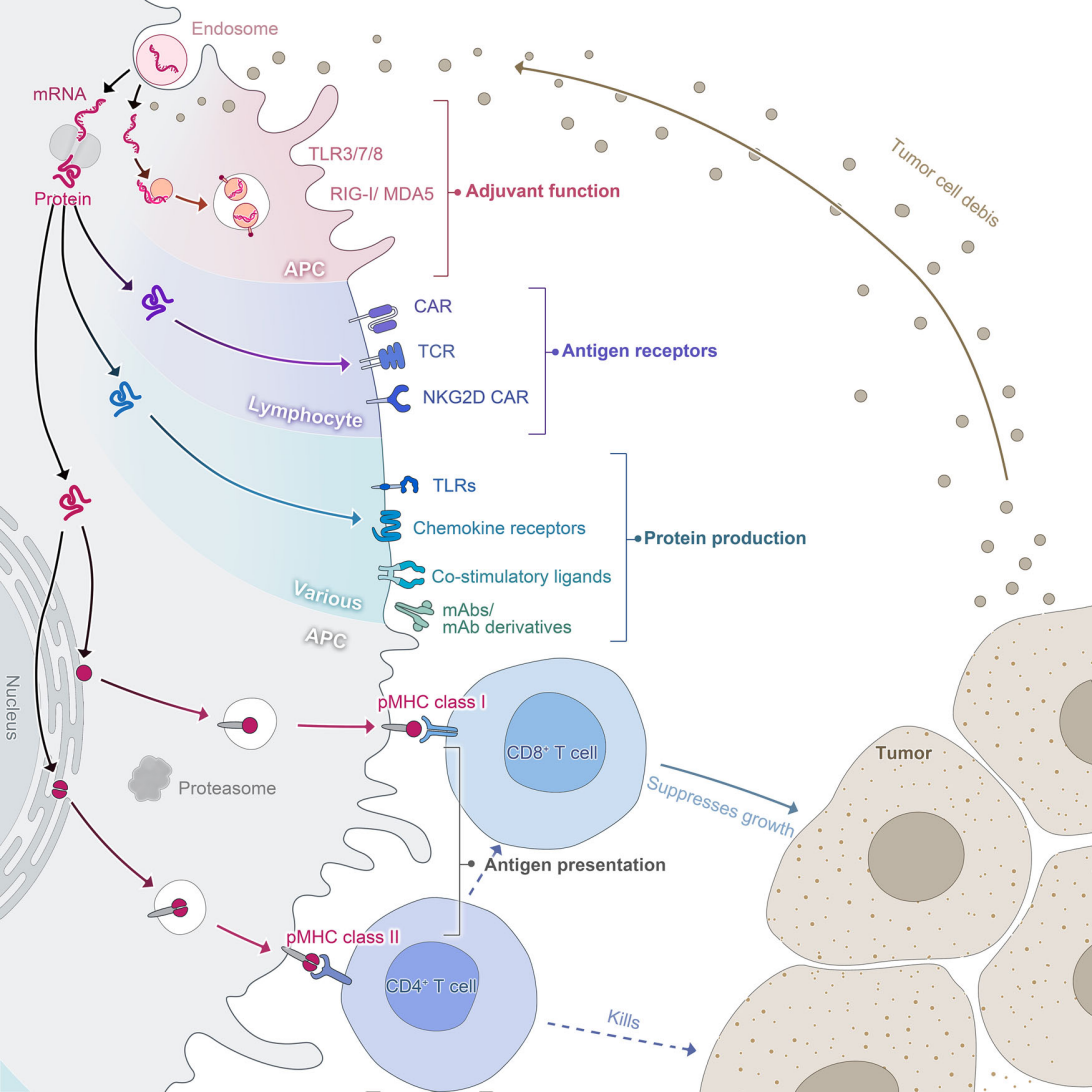
The role of mRNA vaccines in tumour immunity.

There are several polymer‐based vectors, such as poly(l‐lysine) (PLL), poly(amido‐amine) (PAA), poly(beta amino‐esters) (PBAEs) and poly(ethylenimine) (PEI), whereas, only PEI has been widely used for mRNA vaccine delivery.^158^, ^159^ Although PEI offers high gene transfection efficiency, its severe cytotoxicity limits its application, so it is often modified by fatty chains.^160^, ^161^ A novel lipid‐containing polymer called charge‐altering releasable transporters (CARTs) has been explored to effectively deliver mRNA molecules. mRNA molecules and the synthetic Toll‐like receptor‐9 agonist CpG can be encapsulated into a nanoparticle complex by CARTs, then the antigen‐coding mRNA can be safely delivered to antigen‐presenting cells (APCs). After delivery, mRNA is validly translated, processed and presented by MHCs. Also, the codelivery of mRNA and TLR by CARTs simultaneously transfect and activate target cells to motivate an immune response that can clear established tumours in mice.^162^ A study has also found that the mixed‐lipid CARTs are more effective in transfecting lymphocytes, CD4 T cells and CD8 T cells than single‐lipid CART.^163^ Furthermore, polymers are capable of building scaffolding for mRNA vaccination as well. Studies have proved that scaffold‐based mRNA delivery stimulates antigen‐specific antibody in mice^164^, ^165^ (Figure 7).

Although less explored, a peptide‐based delivery method can also be used. The tight combination of mRNA with protamine provides strong protection of mRNA from being degraded by RNases, as well as induces intense immune responses of various immune cells like DCs, monocytes and B cells.^166^, ^167^, ^168^ The clinical trials of the protamine‐formulated mRNA delivery system have proved its great therapeutic effects in a variety of diseases, such as rabies and non‐small cell lung cancer^169^, ^170^ (Figure 6). Cell‐penetrating peptides (CPPs) served as a new delivery approach that promotes the immune reaction of T cells in vivo,^171^ regulates innate immune response and strengthens protein expression in DCs and human cancer cells by facilitating mRNA release from the endosomes and thereby allow expression of mRNA inside the DCs cytosol.^172^ Anionic peptides can increase cell uptake without causing cytotoxicity in DCs through activating both endosome and cytosolic pattern recognition receptors (PRRs) and inducing markers of adaptive responses in primary human DCs in vitro, with prevalent Th1 signature.^173^ Another study has developed a ferritin nanoparticle vaccine to deliver PreS1 to specific bone marrow cells, which induces a durable anti‐PreS1 response and eradicates HBV in mice.^174^

Virus‐like particles (VLPs) use viruses as the carrier to express or present antigens, including poxvirus, adenovirus and herpesvirus, however, merely replication‐defective viruses or attenuated viruses are adopted due to safety reasons.^175^, ^176^ Replication‐defective viruses perform a virus‐infecting manner by encapsulating antigen‐encoding saRNA for delivering into the cytosol, and attenuated viruses sustain the ability of self‐replication.^177^ Compared with the abovementioned delivery systems, VLPs have been successfully applied to promote cancer immunotherapy benefiting from safety and a simple preparation approach. VLPs can be delivered through intradermal inoculation which precludes vaccine particles from leaking into non‐lymphatic organs such as the liver. Also, VLPs reduce the burden of combination therapy with a checkpoint antibody.^178^, ^179^, ^180^ A study designed an mRNA virus‐mimicking vaccine platform using a phospholipid bilayer encapsulated with a protein–nucleotide core consisting of antigen‐encoding mRNA molecules, unmethylated CpG oligonucleotides and positively charged proteins, which offers a potent platform for therapeutic mRNA vaccines confirmed both in vivo and in vitro^181^ (Figure 6).

Other delivery systems include extracellular particles and microneedles. Extracellular particles can be used as an efficient delivery platform based on their physiochemical characteristics, high bioavailability and low non‐targeted cytotoxicity. Exosomes are most used in cancer vaccine development, which exerts dendritic cell‐released major histocompatibility complex (MHC) class I/peptide complexes for efficient CD8^+^ T cell priming to suppress tumour growth.^182^, ^183^ Microneedles, both solid microneedle patches and hollow microneedles for intradermal injection, not only simplify vaccine distribution and improve patient compliance but stimulates the immune responses of the skin. Moreover, microneedles coated with VLPs can elicit stronger immune responses and enable dose sparing compared to intramuscular injection in mice.^184^, ^185^

## 4 LIMITATIONS OF mRNA CANCER VACCINE

Although mRNA vaccines in tumours have multiple advantages, they are still in the initial stage. General side effects, biosafety and limitations of mRNA vaccine require special consideration.

### 4.1 General side effects of mRNA vaccine

The side effects of mRNA vaccine are widely observed in COVID‐19 pandemic. Up until this review, there have been no serious side effects identified for COVID‐19 mRNA vaccines.^186^, ^187^, ^188^, ^189^

### 4.2 Biosafety of mRNA vaccine

#### 4.2.1 Biosafety of physiological disposition

The innate immunity is supposed to be properly activated in order to initiate the adaptive immune response, at the same time, averting the toxic overactivations, which inhibit antigen protein expression as well as immune response. Innate immune response is usually activated by host immune system through pathogen‐associated molecular patterns (PAMPs) from PRRs, the detecting exogenous motifs.^47^, ^190^ However, innate immune sensing of RNAs may dampen the immune response, because of the association with inhibition of antigen expression. Specifically, phage RNA polymerases produce dsRNA that is not desired, which may activate innate immunity through PKR, causing the phosphorylation of eIF‐2, which can block mRNA translation.^190^ In addition, the dsRNA activates RNase L upon binding to OAS,^191^ leading to degradation of the exogenous RNAs. Moreover, dsRNA bound with MDA‐5 and TLR‐3 can activate type I IFN. Therefore, several other genes are elicited and inhibit the translation of mRNA.^192^ Nevertheless, when mRNA structure is improperly designed, PRRs may also be activated, thus abolishing antigen expression (Figure 7).

#### 4.2.2 Biosafety in various crowds

To accelerate the vaccine development, the population enrolled in trials may be limited than expected. It becomes a concern when the vaccine is designed for people throughout the world, for unknown side effects may emerge in the larger population. Certain groups have specific conditions. In older individuals, a different vaccine formulation or a booster dose is supposed to improve immune responses.^193^ In children, as they usually show increased reactogenicity compared to adults, low‐dose vaccines might be required, particularly for mRNA‐based vaccines. In pregnant women, the data on the mRNA vaccine is limited, which makes it difficult to expect whether an equivalent immunological response occurs.^194^ Furthermore, some evidence suggests that babies suffering enduring adverse consequences may be related to variant CD4^+^ T cell responses in their mothers.^195^ In patients on immunosuppressive therapy, immune response to vaccinations is attenuated, which deserves special consideration.^196^

### 4.3 Other limitations of mRNA vaccine

#### 4.3.1 Limitations in type

Although some kinds of bacteria and parasites may cause cancer, researches on mRNA vaccines against bacteria and parasites are rare. It can hardly find antigens in the reproduction cycle of bacteria and parasite which can be made into vaccines. Moreover, some bacteria and parasites have the ability to escape from immunity, at the same time, effective but cheap anti‐bacterial and anti‐parasitic drugs have existed. Therefore, vaccines are intended to fail when assessed by cost/benefit ratio.^197^, ^198^

#### 4.3.2 Limitations in effectiveness

The vaccination program's effectiveness depends on convincing efficacy and safety data accompanied by popular public acceptance and inoculation.^199^ Vaccine immunogenicity and efficacy depend on the points including packaging, storage, preparation and administration. The risks of improperly performing supply chains are detrimental to the safety and effectiveness of the vaccines, with latent consequences of adverse events.^200^ Moreover, vaccine hesitancy remains a noteworthy challenge, which has been described as a ‘lack of confidence in vaccination and/or complacency about vaccination’, which may result in deferment or failure to vaccinate.^201^ There are also worries that the political pressure may hasten the development and approval processes, resulting in an ineffective vaccine being released to the public.^202^

Taken together, the safety of mRNA cancer vaccines is associated with their ability to encode multiple antigens simultaneously as well as being non‐integrating, highly degradable and having no insertional mutagenic potential. In order to develop a safe and effective mRNA vaccine, pre‐clinical trials must be done with caution to avoid severe adverse events. Even after approval, long‐term safety and efficacy data are required. Moreover, cooperation between international organizations provides solid funding insurance for mRNA vaccines.

### 4.4 The future of mRNA cancer vaccine

mRNA‐based cancer vaccines are a rising star with the potential to be versatile, potent, scalable, precise, inexpensive and cold chain free.^51^ These vaccines can avoid several issues associated with DNA vaccines and can be easily manufactured on a large scale for clinical application. A plethora of clinical trials for cancer therapies have demonstrated an increased interest for companies to release mRNA‐based cancer vaccines to the market, which requires a sustainable and cost‐effective manufacturing process that benefits from solving three major concerning issues of instability, innate immunogenicity and inefficient in vivo deliver of mRNA^203^ (Figure 6). As we discussed above, appropriated mRNA structure modification technologies such as nucleotide modifications and codon optimisations, and novel purification methods may foster the efficacy of mRNA internalization by APCs. Moreover, the innate immunogenicity of mRNA can be functioned as an adjuvant‐like effect to boost immune response, however, the paradoxical quality of intrinsic immunity by interferon related pathways may elicit mRNA degradation, leading to less antigen expression.^190^, ^204^ As for promoting delivery efficacy, innovative formulation approaches such as LNPs, CARTs and CPPs show great potential. Therefore, to optimise the mRNA sequence, the purity of mRNA products, the delivery system and administration routes are important for activation of a proper immune response without inducing toxic overactivations.

The anti‐tumour immunity of mRNA‐based cancer vaccines elicits antibody, B cell‐mediated humoral reaction and CD4^+^/CD8^+^ T cells response.^205^, ^206^ There are mainly three types of RNAs currently exploited as cancer vaccines: non‐replicating unmodified SAMs, modified SAMs and virus‐derived SAMs,^203^ among which, SAMs are most investigated in both cancer and infectious diseases because of sustainable effect and frugal dosage.^207^ SAMs are generated from positive single‐stranded mRNA viruses.^208^ They can self‐amplify for up to 2 months and result in a more powerful and long‐lasting immune response, meanwhile, the SAMs platform induces a huge amount of antigen production in the persistent period with lower required dosages of vaccination.^17^ Although SAMs have gained extensive attention and have been an inspiring alternative to mRNA‐based vaccines, clinical applications for cancer treatment are only limited to early assessment of viral replication particles.^209^ With the discovery of neoantigens, personalized vaccines combined with checkpoint blockade modulators or cytokine cocktails are popularized to boost the host anti‐tumour immunity and facilitate the likelihood of tumour cell eradication.^210^, ^211^ Personalized neoantigen‐based cancer vaccines served as tumour‐specific therapies, compared to tumour‐associated antigens, neoantigen‐specific T cells are likely to survive during the progression of immune self‐tolerance, which augments robust T cell response and intensifies the breadth and diversity of the response. Moreover, such vaccines target diverse types of variant mutations to avoid off‐target effects, enhancing the safe quality.^212^ Moderna company invented an mRNA‐based personalized cancer vaccine which composed is of a patient's unique tumour neoantigens. An mRNA‐based vaccine that targets each of these mutations, 20 neoepitopes which represent mutations of the patient's cancer cells were predicted, was injected into the patient. The vaccine is able to organize the patient's cells which express the specific neoepitopes and help the immune system differentiate cancer cells from normal cells. As results, the patient's immune system can better distinguish the cancer cells and eliminate them. Additionally, combining mRNA‐based cancer vaccines with other therapies can enhance anti‐tumour effects (Figure 6). In 2016, Moderna company and Merck company intended to combine mRNA‐4157 which is associated with checkpoint inhibitor therapies with anti‐PD‐1 therapy, KEYTRUDA. Currently, a phase I study has been conducted to investigate the safety, tolerability and immunogenicity of mRNA‐4157 alone in subjects with resected solid tumours, and the combination strategy with KEYNOTE‐603 in subjects with unresectable solid tumours.^213^ Two phase II studies by BioNTech evaluate the efficacy tolerability and safety of the mRNA‐based cancer vaccines in treating patients with anti‐PD‐1‐refractory/relapsed unresectable stage III or IV melanoma and colorectal cancer patients who underwent surgery and chemotherapy, respectively.^214^, ^215^ BNT111 in combination with Libtayo is expected to activate a potent and precise immune response against cancer,^214^ while BNT122 is considered a precision medicine for colorectal cancer patients, tailored to match personal tumour characteristics for an individual subject, resulting in more cost.^215^

The instability of vaccine formulations hinders the distribution of the vaccine in remote, rural areas, which is most associated with temperature‐dependent degradation in that high temperatures accelerate many destabilization pathways, causing millions of people to die from vaccine‐preventable diseases. For this reason, cold chain systems have been developed for the manufacturing, transportation, storage and distribution of vaccines. Lyophilization, freeze‐drying, is one of the most common methods for long‐term preservation of vaccines. Lyophilization provides drying without damaging antigens as well as allows for an aseptic process to meet sterility requirements. However, the lyophilization needs to be reconstituted and typically used within a few hours when administrating parenterally, leading to more difficult administration. Also, water‐mediated destabilization pathways can be increased by reconstitution, resulting in the reduction of potency of lyophilized vaccines.^216^ Spray drying is an alternative to producing dried vaccines due to its low cost and less energy consumption compared with lyophilization.^217^ However, new technologies should be developed to stabilize vaccines while alleviating some of the limitations of traditional lyophilized and spray drying.

Taken together, although mRNA cancer vaccines show great anti‐tumour potential, a lot of issues remain to be solved to promote the efficacy, such as immunogenic platforms, antigens, adjuvants, doses, delivery materials, prime/boost strategies, frequency and routes of administration (Figure 6). Moreover, few studies have directly compared the efficacy of various vaccines, which should be a critical area of future research.

## 5 CONCLUSION AND PERSPECTIVES

In conclusion, the COVID‐19 pandemic has led to the considerable development of mRNA vaccines in both cancer and infectious diseases fields. In comparison with the prophylactic vaccines for infectious diseases, most cancer vaccines are therapeutic. There are only two prophylactic cancer vaccines approved by FDA, which are HBV and HPV vaccines to prevent virus‐induced hepatocellular carcinoma and cervical cancer, respectively. The therapeutic cancer vaccines are positive systemic immunotherapies that intensify anti‐tumour immunity via activating and expanding antigen‐specific CD4^+^ and CD8^+^ T cells. With the innovation of technologies, mRNA‐based cancer vaccines present higher efficacy compared to other types of cancer vaccines, since mRNA can simultaneously encode multiple antigens, or enhance antibody, humoral immune response and cellular adaptive immune response. Moreover, formulation platforms and the manufacturing process of mRNA vaccines are relatively mature, which allows to create or produce cancer vaccines rapidly and abundantly. Furthermore, the introduction of neoantigens encourages personalized mRNA‐based cancer vaccines applications, providing more precise and potent anti‐tumour effects. The goal of using mRNA cancer vaccines is supposed to maximize efficacy while minimizing the adverse effects. Although there are many advantages of the mRNA vaccine, further improvements in the manufacturing processes, and a more comprehensive understanding of the mechanisms of action in multiple mRNA vaccine types are necessary. The goal of using mRNA cancer vaccines is supposed to maximize efficacy while minimizing the adverse effects.

## CONFLICT OF INTEREST STATEMENT

The authors declare no conflicts of interest.
